# Study on the skin structure, hair follicle cycle, and GSDMA protein expression in Ganxi goats

**DOI:** 10.3389/fvets.2025.1661505

**Published:** 2025-09-22

**Authors:** Xue Yang, Dengxiang Ji, Caiyan Wen, He Chen, Zhangyong Jin, Luyun He, Lucheng Zheng, Ben Liu, Qingcan Fan, Wei Hu, Wenya Zheng, Qianqian Wang, Yan Hu

**Affiliations:** ^1^College of Life Science and Resources and Environment, Yichun University, Yichun, China; ^2^Engineering Technology Research Center of Jiangxi Universities and Colleges for Selenium Agriculture, Yichun University, Yichun, China; ^3^Yichun University Research Center for Traditional Chinese Veterinary Medicine and Animal Embryo Engineering Technology, Yichun University, Yichun, China; ^4^Traditional Chinese Medicine Room, Yichun Second People's Hospital, Yichun, China

**Keywords:** Ganxi goats, skin, melanin, proliferating cells, hair follicle cycle, GSDMA

## Abstract

**Introduction:**

The Ganxi goat, a native Chinese breed inhabiting the hot and humid regions of western Jiangxi Province, displays notable adaptability to local climatic stress. This study aimed to investigate the morphological structure, hair follicle cycling pattern, and GSDMA protein expression in the skin of Ganxi goats, to elucidate the potential mechanisms underlying their environmental adaptation.

**Methods:**

Using histological (H&E, Sacpic, and melanin staining), immunohistochemical (Ki67 and GSDMA), and Western blotting techniques, we conducted a year-long analysis of skin samples from ten 6-month-old female Ganxi goats.

**Results:**

Results showed that total skin thickness ranged from 1,118 to 2088 μm, and epidermal thickness from 12 to 28 μm, with regional variation. Primary hair follicle depth averaged 1,056 μm. Hair follicle groups exhibited a typical trimeric structure, but were looser than in other goat breeds, with less-developed connective tissue sheaths, wider inter-follicular spaces, and well-developed sweat glands. Melanin was mainly localized in hair bulbs and outer root sheaths. Ki67-positive cells were concentrated in hair matrix regions. These structural features suggest that Ganxi goat skin is morphologically adapted to hot and humid environments. The secondary hair follicle cycle was divided into four phases: anagen (October–February), late anagen (March), catagen (April–August), and telogen (September). Ki67-positive cells were mainly located in the hair matrix, outer root sheath, and sebaceous glands, indicating active cell proliferation. Melanin was primarily distributed in the hair bulb and outer root sheath, but absent in the epidermis. GSDMA protein was cytoplasmically expressed in the epidermis, hair follicles, and sebaceous glands, with its level peaking in late anagen and decreasing through catagen and telogen phases.

**Discussion:**

These findings highlight the structural and molecular adaptations of Ganxi goat skin to hot and humid environments and suggest that GSDMA may be involved in regulating the hair follicle cycle and maintaining skin homeostasis, and that further functional studies are required to establish a direct role in environmental adaptation.

## 1 Introduction

The skin, as the outermost structure of the animal body, serves multiple vital physiological functions, including acting as a protective barrier, facilitating sensory transmission, regulating body temperature, and participating in immune responses (1). The morphological characteristics of its tissue structure largely reflect an animal’s adaptability to environmental conditions. Particularly under extreme climatic conditions such as high temperature and humidity, structural differences in the skin significantly influence the organism’s ability to resist heat stress and maintain homeostasis (2). Hair follicles are important appendage structures of the skin, and their cyclical activity directly influences hair growth, shedding, and regeneration. The hair follicle growth cycle primarily consists of three phases: anagen, catagen, and telogen (3, 4), which are accompanied by dynamic changes such as cell proliferation, differentiation, and apoptosis within the hair follicle (5–7). Factors such as the number and distribution of hair follicles, the thickness of the epidermis and dermis, the development of sweat and sebaceous glands, and the deposition of melanin are all closely associated with the efficiency of heat exchange and the ability of animals to respond to external stimuli (8, 9).

Yichun, located in the western region of Jiangxi Province in southeastern China, falls within a subtropical humid monsoon climate zone. Summers in this region are characterized by high temperatures and humidity, resulting in a high level of heat load. Under such environmental conditions, animals face considerable risk of heat stress and must adapt to the hot and humid climate by enhancing heat dissipation and adjusting skin structure and function. As a key organ for heat exchange, the regional structural characteristics of the skin form part of the physiological foundation developed by animals through long-term ecological adaptation (10, 11).

The Ganxi goat is a local Chinese breed widely distributed across the hilly and mountainous regions of western Jiangxi Province. It exhibits strong adaptability to environmental conditions. Having lived in a hot and humid ecological environment over the long term, this breed’s skin may possess specific structural adaptations that enhance heat dissipation efficiency and improve the animal’s tolerance to thermal stress. However, studies on the skin tissue structure of Ganxi goats remain limited, particularly with respect to the systematic investigation of its structural characteristics, cellular proliferative activity, and the developmental patterns of the hair follicle cycle.

Gasdermin A (GSDMA) is an epithelial protein implicated in keratinocyte differentiation (12), barrier integrity (13), and inflammatory skin responses (14). Altered GSDMA expression has been linked to disorders such as psoriasis (15) and alopecia (16), suggesting a role in follicle biology. Ki67, a nuclear antigen expressed during active phases of the cell cycle, is widely used to evaluate proliferative activity in epidermis and hair follicles (17). Together, these markers provide a framework to explore the cellular and molecular dynamics of Ganxi goat skin.

In this study, Ganxi goats were selected as the research subjects. A systematic analysis was conducted using histochemical staining (H&E, Sacpic, and melanin staining), immunohistochemical staining (Ki67 and GSDMA), and Western blotting (GSDMA) to investigate skin thickness, the structure of hair follicle groups, melanocyte distribution, localization of proliferating cells, preliminary classification of the secondary hair follicle cycle, and the expression patterns of GSDMA during different hair follicle stages. The aim was to reveal the adaptive characteristics of Ganxi goat skin under hot and humid climatic conditions, and to provide a theoretical basis for studies on the ecological adaptation mechanisms of this local breed as well as for the development and utilization of its skin and hair resources.

## 2 Materials and methods

### 2.1 Experimental animals and sampling

Ten healthy 6-month-old female Ganxi goats were purchased from Yuanzhou District, Yichun City. During the experimental period, the animals were allowed ad libitum access to feed. Skin tissue samples were collected monthly for 1 year from the lateral neck region of each goat and divided into two portions. One portion was fixed in 4% paraformaldehyde solution prepared in pre-cooled 0.1 M phosphate-buffered saline (PBS) at 4°C, and the other portion was snap-frozen in liquid nitrogen and stored at −80°C for subsequent analysis. At the final sampling point, when the goats reached 18 months of age, they were euthanized via exsanguination from the carotid artery. Immediately after skinning, skin samples from various anatomical regions were collected, and the total skin thickness was measured using a digital caliper. Measurements were repeated five times at each site. The samples were labeled and fixed in 4% paraformaldehyde prepared with pre-cooled 0.1 M PBS at 4°C for further histological processing. All experimental procedures involving animals were approved by the Animal Ethics Committee of Yichun University (No. LSK2022-028).

### 2.2 Reagents and instruments

Hematoxylin–eosin (H&E) staining kit, Sacpic staining kit, Masson-Fontana melanin staining kit, and high-efficiency RIPA tissue/cell lysis buffer were purchased from Solarbio Life Sciences (Beijing, China). 5 × protein loading buffer, rabbit anti-β-actin antibody, and protein marker were obtained from Servicebio Technology (Wuhan, China). 10 × RealBlot rapid transfer buffer was purchased from BaiSha Biotech (Hefei, China). The GSDMA antibody was purchased from Bioss Biotechnology (Beijing, China), and the Ki67 antibody was obtained from Abcam (USA). The HRP-conjugated goat anti-rabbit secondary antibody was purchased from ImmunoWay (USA), and the protein quantification kit was obtained from Pulilai Biotechnology (Beijing, China). The immunohistochemistry (IHC) detection kit and DAB chromogenic kit were purchased from Zhongshan Golden Bridge Biotechnology Co., Ltd. (Beijing, China).

A paraffin microtome was purchased from Kedi Instrument Equipment Co., Ltd. (Jinhua, Zhejiang, China). The biological microscope (Olympus BX53 with DP73) and imaging system were obtained from Olympus (Japan), and the multifunctional imaging system was obtained from GE Healthcare (USA).

### 2.3 Methods

#### 2.3.1 Tissue section preparation

The Ganxi goat skin samples were fixed for more than 48 h. Square tissue sections with a side length of 0.5 cm were cut, ensuring that the hair direction was noted, with one side of the tissue parallel to the hair growth direction. The samples were then washed with tap water for 24 h, followed by softening in a 1:1 mixture of alcohol and glycerol for 24 h. The dehydration process was carried out as follows: 70% alcohol for 6 h, 80% alcohol overnight, 95% alcohol for 2 h (repeated twice), and 100% alcohol for 1 h (repeated twice). The samples were then cleared in xylene for 10 min (repeated twice). For wax infiltration, the tissues were immersed in low-melting paraffin for 2.5 h, medium-melting paraffin for 1.5 h, and high-melting paraffin for 1 h. Finally, the tissue samples were embedded and sectioned into 7 μm continuous transverse and longitudinal sections.

#### 2.3.2 HE staining

The skin tissue sections were dewaxed with xylene for 10 min, repeated twice. They were then rehydrated through a graded ethanol series: 100% ethanol for 10 min (repeated twice), 95% ethanol for 5 min (repeated twice), 80% ethanol for 5 min, 70% ethanol for 5 min, and distilled water for 5 min. Hematoxylin staining was performed for 5 min, followed by differentiation with acid alcohol for 10 s. Sections were blued under running tap water for 15 min, then counterstained with eosin for 2 min. After dehydration through a graded ethanol series and clearing with xylene, the sections were mounted with neutral resin. The thickness of the epidermal layer and the depth of primary hair follicles were observed, photographed, and measured under a microscope (magnifications: 100×, 200×, 400×).

#### 2.3.3 Sacpic staining

The skin tissue sections were deparaffinized in xylene, followed by gradient alcohol rehydration. Then, the sections were stained with Azure Blue B solution for 5 min, rinsed with distilled water for 5 s, and stained with Weigert’s Hematoxylin solution for 5 min. After another rinse in distilled water for 5 s, the sections were returned to blue by washing with blueing water for 3 min. Next, the sections were stained with Sand Yellow solution for 5 min, differentiated in 70% ethanol for 3 s, and further differentiated in 95% ethanol for 3 s. Then, they were stained with Picroacetic Ethanol solution for 3 min, followed by differentiation in 95% ethanol for 3 s and 70% ethanol for 3 s. After a final rinse in distilled water for 5 s, the sections were stained with Indigo Carmine solution for 1 min, rinsed again with distilled water for 5 s, dehydrated through gradient alcohol, cleared in xylene, and mounted with neutral balsam. The slides were observed and photographed under a microscope (magnifications: 100×, 200×, 400×). The assignment of months to follicle phases was based on repeated histological observation of monthly samples. Criteria followed Nixon (18) and Welle (19): intact inner root sheath and large bulb (anagen), beginning regression of bulb and partial sheath breakdown (late anagen), shaft shedding and follicle shrinkage (catagen), and absence of sheath with regressed follicle cavity (telogen).

#### 2.3.4 Masson-Fontana melanin staining

Skin tissue sections were prepared according to the instructions for Masson-Fontana melanin staining solution (Solarbio, China). The sections were deparaffinized in xylene, followed by gradient alcohol rehydration. Then, the Fontana ammoniacal silver solution was applied, and the sections were incubated at 56°C for 30 min in the dark. After incubation, the sections were rinsed in distilled water for 2 min, repeated 6 times. The sections were then treated with Hempel’s solution for 3 min, followed by a 5-min rinse in tap water. The sections were counterstained with neutral red for 5 min, rinsed in distilled water for 1 min, dehydrated, cleared in xylene, and mounted with neutral balsam. The slides were observed and photographed under a microscope (magnifications: 100×, 200×, 400×). To ensure the accuracy of melanin localization, serial sections were prepared from the same follicular region. The position of the section was determined based on cellular morphology within the follicle: a cup-shaped or annular structure with aggregation of dermal papilla and matrix cells was identified as the hair bulb, whereas a homogeneous, acellular structure corresponded to the subcutaneous hair shaft, i.e., the region above the bulb. By comparing serial sections and staining results, we were able to clearly distinguish the hair bulb from the hair shaft region, thereby accurately determining the sites of melanin-positive distribution.

#### 2.3.5 Immunohistochemical (IHC) staining

After deparaffinization and rehydration, skin tissue sections were processed according to the instructions of immunohistochemistry detection kit for rabbit primary antibodies. The Ki67 primary antibody was diluted at 1:200, and the GSDMA primary antibody at 1:600. For the negative control, 1 × PBS was used instead of the primary antibody. Color development was performed using a DAB staining kit. The staining reaction was monitored under a light microscope and terminated at the appropriate time with distilled water. Hematoxylin was used for counterstaining, followed by dehydration through a graded ethanol series, clearing in xylene, and mounting with neutral balsam. The stained sections were then observed and imaged under a microscope (magnifications: 100×, 200×, 400×).

#### 2.3.6 Western blot analysis

For each month from January to December, 60 mg of skin tissue was weighed and placed into a 2 ml EP tube. A total of 495 μl RIPA lysis buffer and 5 μl PMSF were added, and the mixture was homogenized on ice. After standing for 30 min, the samples were centrifuged, and the supernatant was collected. Protein concentrations were determined using a BCA protein assay kit. Each 40 μl protein sample was mixed with 10 μl of 5 × loading buffer, boiled at 95°C for 5 min, and stored at −20°C. Total protein samples were separated by 12% SDS-PAGE and transferred onto PVDF membranes using a semi-dry transfer system. Membranes were washed with TBST, blocked with 5% non-fat milk for 2 h at room temperature, and washed again with TBST three times. The membranes were then incubated overnight at 4°C with primary antibodies against GSDMA (1,8,000) and β-actin (1,14,000). After three additional washes with TBST, membranes were incubated with HRP-conjugated secondary antibody (1,10,000) for 90 min at room temperature. Finally, the protein bands were visualized using an enhanced chemiluminescence (ECL) detection kit and a multifunctional imaging system.

### 2.4 Data analysis

For Western blot analysis, the relative expression levels of target protein bands were quantified using ImageJ software. For epidermal thickness and hair follicle depth analysis, three skin sections were selected for each region, epidermal thickness was measured perpendicularly from the basal layer of the epidermis to the stratum corneum at five random non-overlapping fields per section under 400 × magnification. Primary hair follicle depth was measured from the epidermal surface to the base of the follicle bulb. Measurements were performed independently by two observers, and each site was measured five times to ensure reproducibility. For immunohistochemical analysis, three skin sections were selected for each month (from January to December) under a 400 × light microscope, with each section containing at least three non-overlapping fields of view. The average optical density (AOD) of positive staining signals was measured using ImageJ software. Statistical analysis was performed using GraphPad Prism 9.5.0. One-way analysis of variance (ANOVA) was conducted using SPSS 27.0, and Duncan’s multiple range test was used for *post hoc* comparisons. A *p* < 0.05 was considered statistically significant.

## 3 Results

### 3.1 Statistical measurement of skin data in Ganxi goats

**Figure 1:**
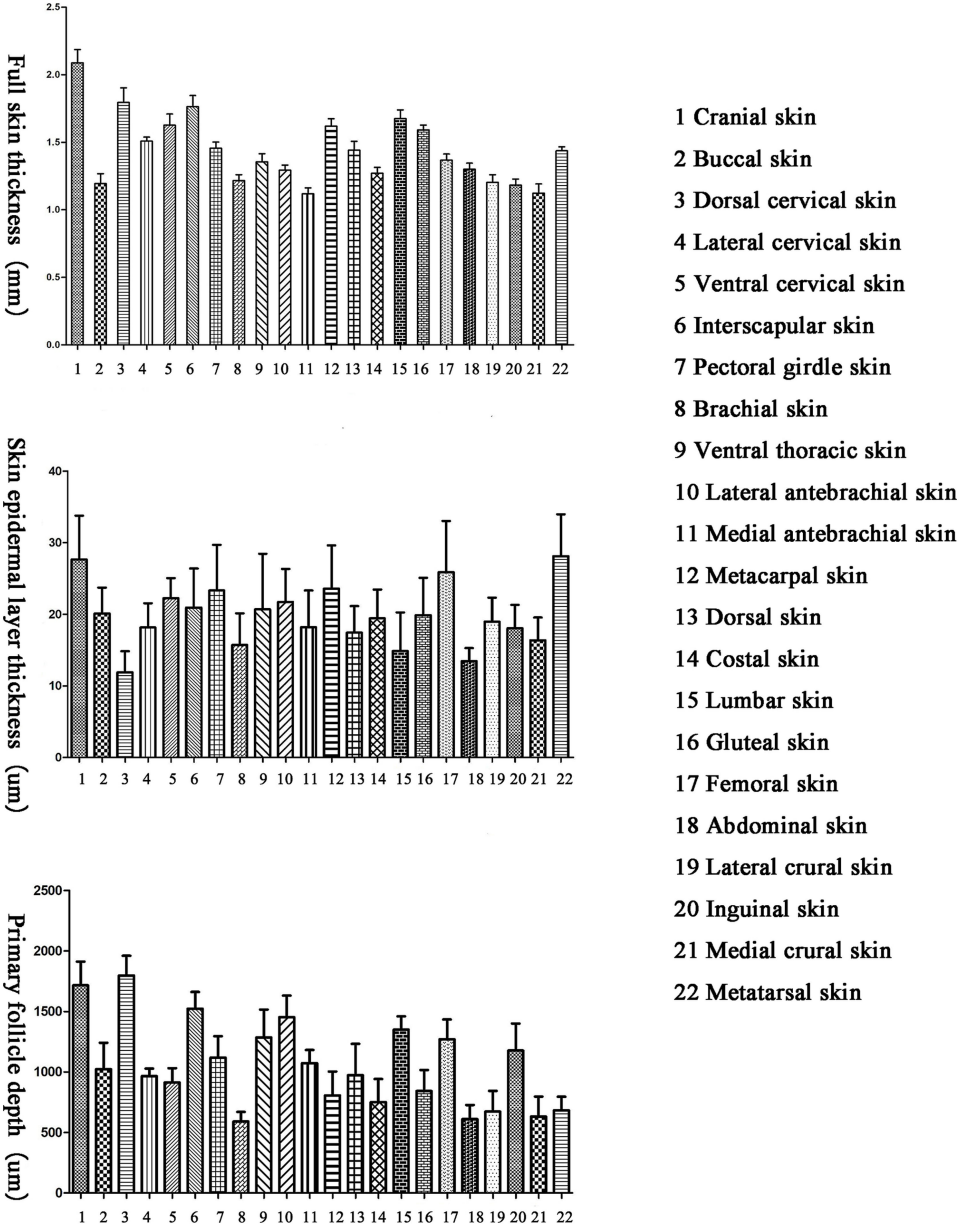
Total skin thickness, skin epidermal layer thickness and skin primary hair follicle depth of different parts of Ganxi goat.

There are differences in the full skin thickness of Ganxi goats across different body regions, with a range of 1,118–2088 μm and an average of 1,438 μm. The thickest skin is found on the cranial region, followed by the dorsal and ventral cervical regions, interscapular, metacarpal, lumbar and gluteal regions, while the thinnest skin is on the medial antebrachial region, followed by the medial crural and buccal regions (Figure 1).

There are differences in the epidermal thickness of Ganxi goats across different body regions, with a range of 12–28 μm and an average of 20 μm. The thickest epidermis is found on the metatarsal region, followed by the femoral and metacarpal regions, while the thinnest epidermis is on the dorsal cervical region, followed by the abdominal and lumbar regions (Figure 1).

There are differences in the depth of primary hair follicles in Ganxi goats across different body regions, with a range of 591–1797 μm and an average of 1,056 μm. The deepest primary hair follicles are found on the dorsal cervical region, followed by the cranial, interscapular, lateral antebrachial, lumbar, and other regions, while the shallowest primary hair follicles are found on the gluteal region, followed by the abdominal and medial crural regions (Figure 1).

### 3.2 Observation of skin structure in the lateral neck region of Ganxi goats

**Figure 2:**
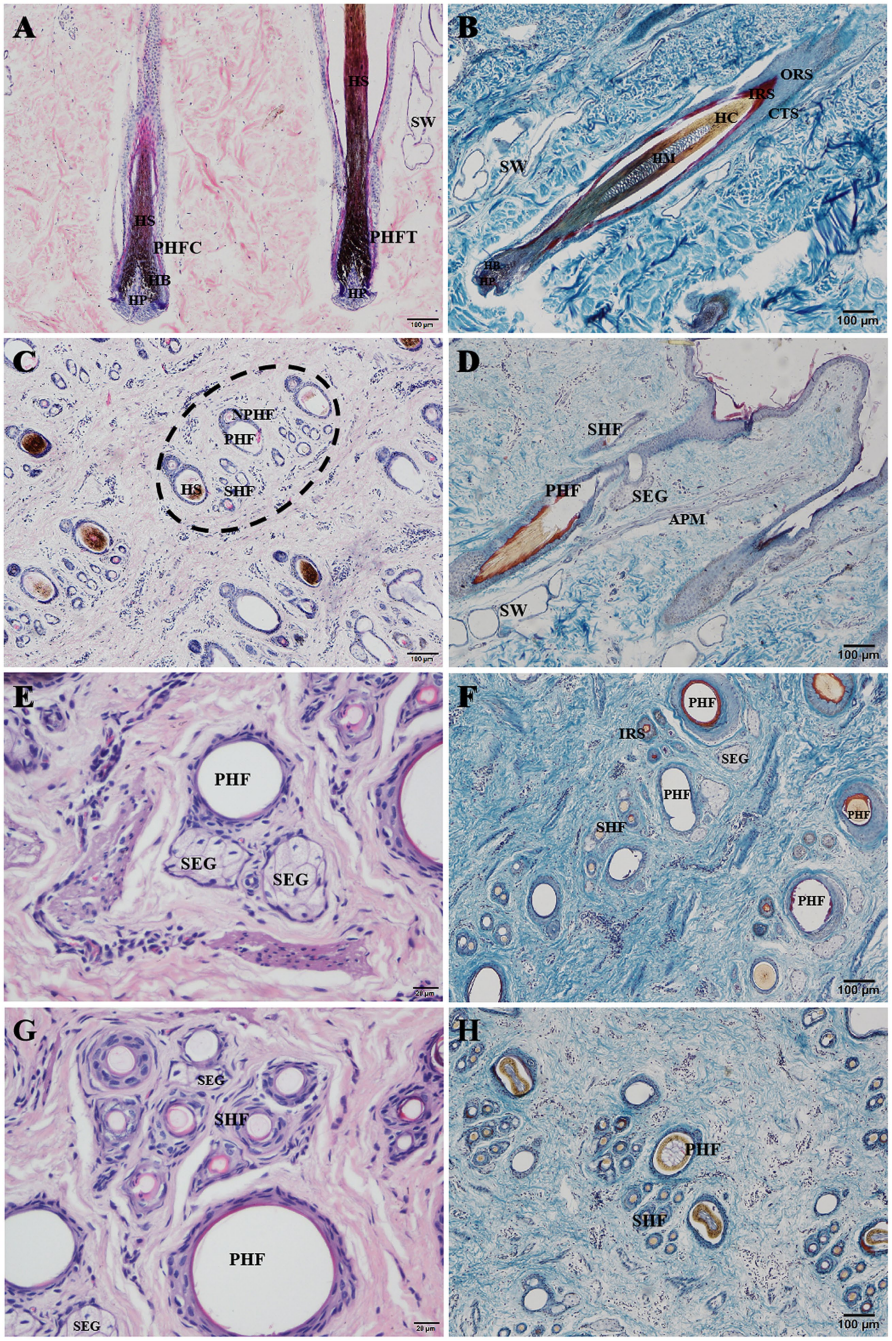
Histological structures of hair follicle in Ganxi goat. **(A)** Primary hair follicle slitting, HE stain, 100×; **(B)** Primary hair follicle, Sacpic stain, 100×; **(C)** The hair follicle group was transected (a complete hair follicle group was in the oval virtual frame), HE stain, 100×; **(D)** Primary hair follicles were cut longitudinally, Sacpic stain, 400×; **(E)** Sebaceous gland, HE stain, 400×; **(F)** The hair follicle group was transected, Sacpic stain, 100×; **(G)** Secondary hair follicles were transected, HE stain, 400×; **(H)** The hair follicle group was transected, Sacpic stain, 100×. PHFC, primary hair follicle in anagen; PHFT, primary hair follicle in telogen; PHF, primary hair follicle; SHF, secondary hair follicle; NPHF, new primary hair follicle; HP, hair papilla; HB, hair bulb; HS, hair shaft; HC, hair cortex; HM, hairy medulla; IRS, inner root sheath; ORS, outer root sheath; CTS, connective tissue sheath; SW, sweat gland; SEG, sebaceous gland; APM, arrector pili muscle.

**Figure 3:**
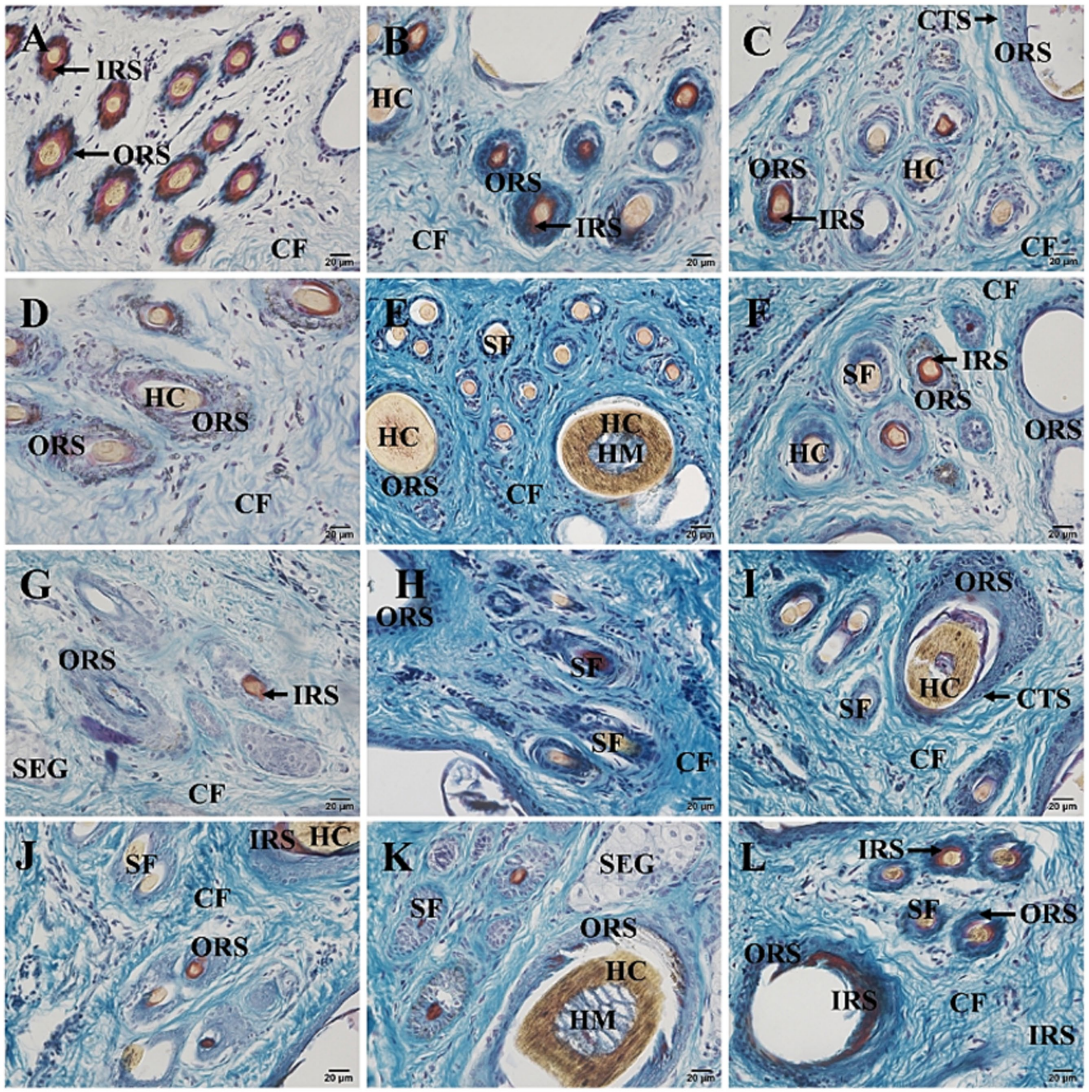
Cross-section of skin tissue of 12-month-old Ganxi goats (Sacpic staining). **(A–L)** The skin cross section of Ganxi goat from January to December, 400×. SF, secondary hair follicles; SEG, sebaceous gland; CF, Collagen fiber; CTS, connective tissue sheath; HM, medulla; HC, hair cortex; IRS, inner root sheath; ORS, outer root sheath.

The hair follicles of Ganxi goats were divided into primary and secondary follicles. Primary follicles were larger in diameter and extended deeper into the dermis, whereas secondary follicles were smaller and more superficially located. Follicle cycle stages were identified using established histological criteria, including the morphology of the hair bulb, the presence or absence of dermal papilla, and Sacpic staining of the inner root sheath (18, 19). In the anagen phase, follicles exhibited large hair bulbs with prominent dermal papillae and a continuous red-stained inner root sheath. During catagen, the bulb regressed, the inner root sheath became shortened and fragmented, and the hair shaft began to detach. In telogen, follicles showed absence of the inner root sheath and contained empty or keratinized hair shafts. Primary follicles often cycled independently, with adjacent follicles in different phases within the same group (20). In contrast, secondary follicles exhibited more synchronized cycling: they were abundant and active in anagen, gradually regressed in catagen, and became largely quiescent in telogen, although occasional activity persisted even in telogen. Primary and secondary hair follicles are regularly combined into hair follicle groups, which are oval in shape and arranged in a regular pattern within the skin. The intervals between hair follicle groups range from 0.5 to 1 of their own width and are primarily composed of dense connective tissue and stromal cells. Each hair follicle group typically consists of 3 primary hair follicles arranged in a straight line, with 10–20 secondary hair follicles distributed in two groups on one side of the primary hair follicles. Each group of secondary hair follicles is located between two primary hair follicles. The arrangement of hair follicle groups in the same area follows a consistent pattern, as shown in Figure 2C. Some hair follicle groups may also contain 2 or 4 primary hair follicles, and the number of secondary hair follicle groups will adjust accordingly. Near the primary hair follicles on the opposite side of the secondary hair follicle group, a round cluster of cells or a hair follicle, tightly adjacent to the primary hair follicle, can be observed, which represents a new primary hair follicle replacing the corresponding old primary hair follicle. In Figure 2A, two adjacent primary hair follicles are observed, one in the anagen phase and the other in the catagen phase. The hair follicle consists of the following layers from the innermost to the outermost: hair (hair medulla, hair cortex), inner root sheath, outer root sheath, and connective tissue sheath, with the skin ending in a hair bulb, which is cup-shaped and accommodates the dermal papilla (Figure 2B). In Figure 2H, one of the three primary hair follicles has a shed hair shaft, leaving an empty hair follicle cavity, one has a hair shaft with medulla, and the other has a shriveled, soon-to-be-shed hair shaft, forming an “8” shape. Both primary and secondary hair follicles are associated with sebaceous glands. The sebaceous glands of the primary hair follicles are well-developed and consist of two lobes (Figure 2E), opening on one side of the primary hair follicle (Figure 2D). The sebaceous glands of the secondary hair follicles are underdeveloped, composed of only a few sebaceous cells located between the secondary hair follicles, and not every secondary hair follicle has a sebaceous gland (Figure 2G). Sacpic staining can turn the inner root sheath of the hair follicle bright red and the outer root sheath blue, making it easier to distinguish and allowing the activity of the hair follicle to be determined based on the presence of the inner root sheath. In the anagen phase, the primary hair follicle shows a bright red inner root sheath (Figure 2G), primarily in the part of the follicle below the sebaceous gland (Figure 2D), while in the catagen phase, the inner root sheath of the hair follicle becomes atrophied, shorter, and incomplete (Figure 2F), and in the telogen phase, the inner root sheath is usually absent (Figure 2F). However, even in the telogen phase, although most secondary hair follicles have lost the inner root sheath, some secondary hair follicles can still show activity (Figure 2F). After Sacpic staining of Ganxi goat skin samples collected over 12 months, the cyclic changes of hair follicles were observed (Figure 3). Based on the morphological changes of hair follicles, the growth cycle of secondary hair follicles in Ganxi goats can be divided into four phases: anagen (October to February of the following year), late anagen (March), catagen (April to August), and telogen (September).

### 3.3 Distribution of melanin in the skin on lateral neck region of Ganxi goats

**Figure 4:**
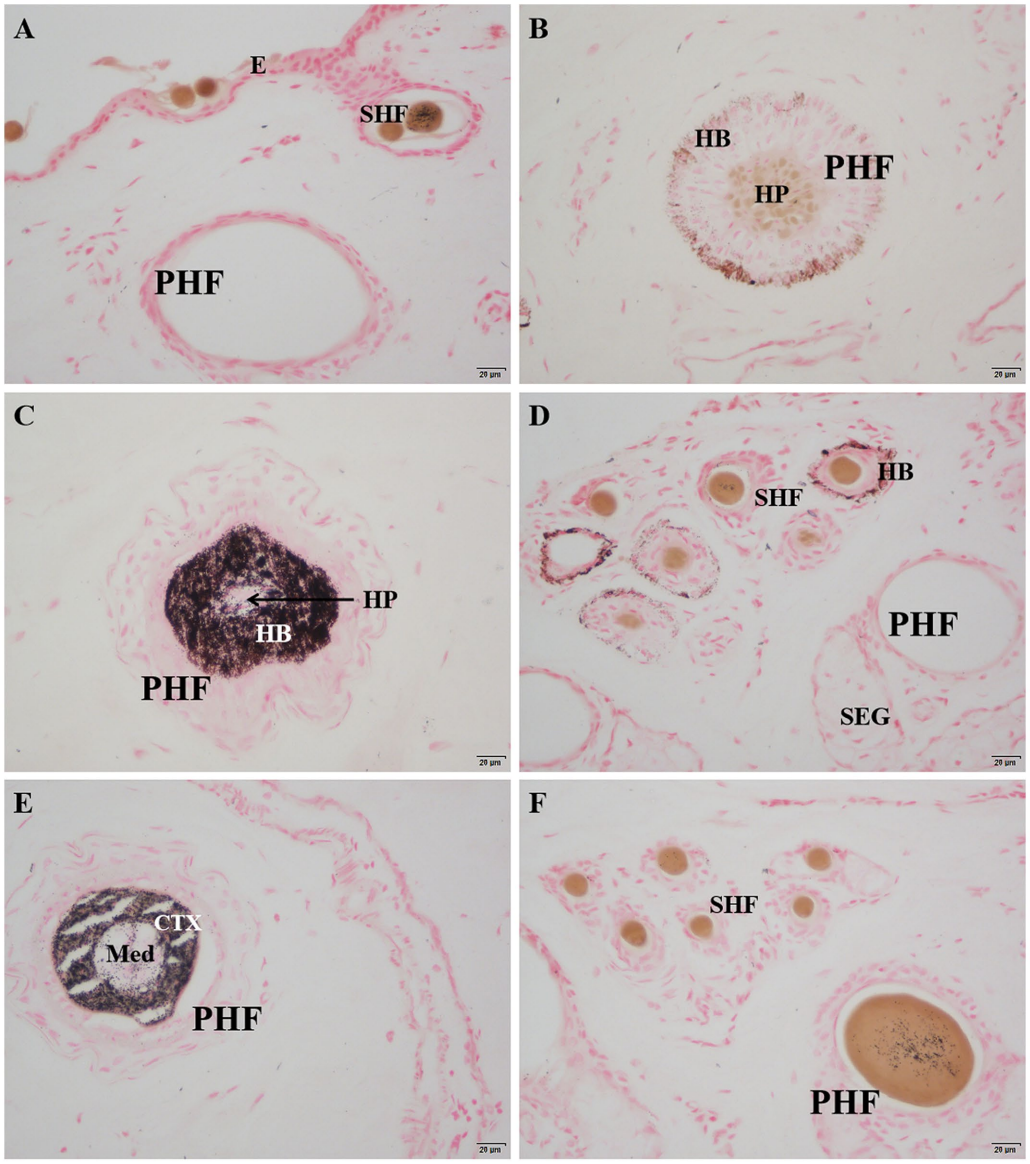
Distribution of Masson-Fontana melanin in the skin of Ganxi goats. **(A)** Epidermis, 400×; **(B)** Lower part of primary hair follicle, 400×; **(C)** Upper part of primary hair follicle, 400×; **(D)** Secondary follicle hairball, 400×. **(E)** Primary follicle hair shaft, 400×; **(F)** Secondary follicle group, 400×. Masson-fontana melanin staining. E, epidermis; PHF, primary hair follicle; SHF, secondary hair follicle; HP, hair papilla; HB, hair bulb; SEG, sebaceous gland; CTX, Cortex of hair shaft; Med, Medulla of hair shaft.

After Masson-Fontana melanin staining, melanin in the skin appeared black, while the cell nuclei were stained pink. The results showed that there was no melanin present in the epidermis (Figure 4A). Melanin was mainly distributed in the hair bulb (Figure 4C), the outer root sheath of both primary and secondary hair follicles (Figures 4B,D), the cortex of the main hair (Figure 4E), and also a small amount in the hair shafts of the lanugo (Figure 4F).

### 3.4 Distribution of proliferating cells in the skin on lateral neck region of Ganxi goats

**Figure 5:**
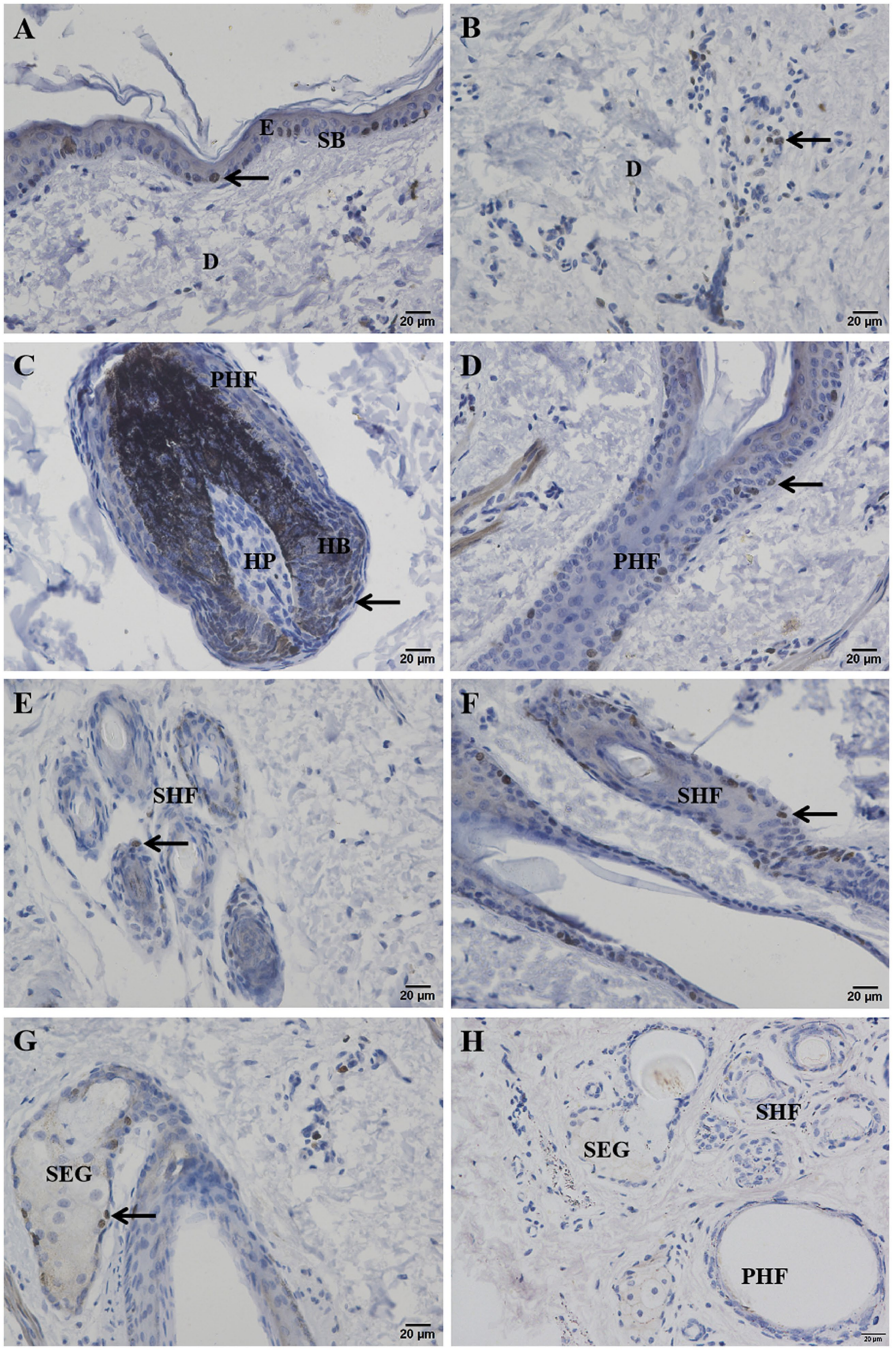
Ki67 positive cells on the distribution of the Ganxi goat skin. **(A)** Epidermis; **(B)** Dermal interstitial cells; **(C)** primary hair follicle hairball; **(D)** infundibular part of primary hair follicle; **(E)** transverse cutting of secondary hair follicles; **(F)** secondary hair follicle slitting; **(G)** sebaceous glands; **(H)** Negative control. Arrows indicate Ki67-positive cells, 400×. E, epidermis; SB, basal layer; D, genuine leather; PHF, primary hair follicle; SHF, secondary hair follicle; HP, dermal papilla; HB, hairball; SEG, sebaceous glands.

**Figure 6:**
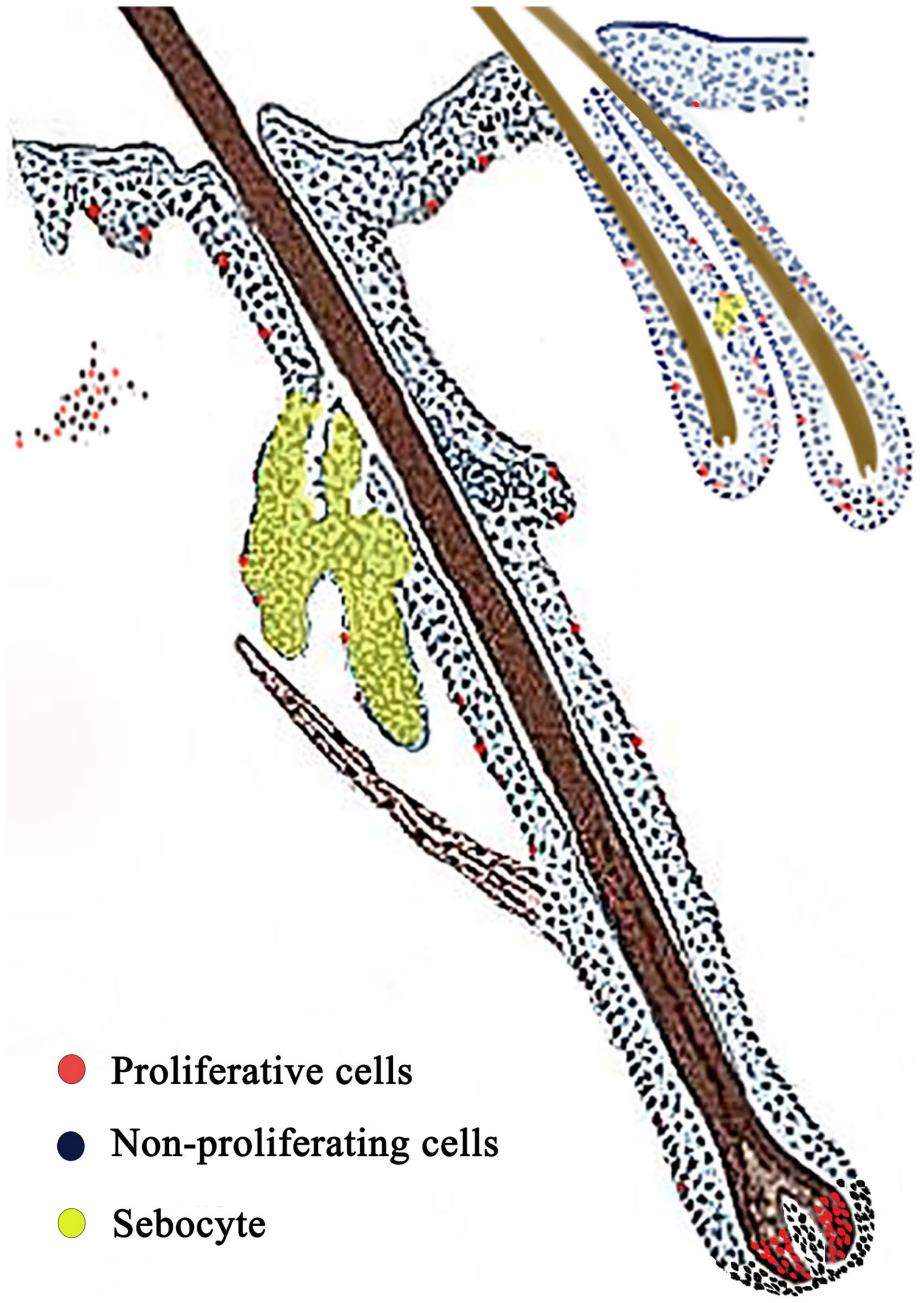
Skin proliferation cell distribution model. Red: proliferating cells; Blue: non-proliferating cells; Yellow area: sebocyte.

Ki67 immunohistochemistry-positive cells, indicating proliferating cells, appeared brownish-black, while the cell nuclei of other cells were stained blue by hematoxylin. Ki67-positive cells were sparsely observed in the basal layer of the epidermis (Figure 5A), dermal stromal cells (Figure 5B), the outer root sheath cells of primary and secondary hair follicles (Figures 5D,E), and the epithelial cells of sebaceous glands (Figure 5G). A significant number of positive cells were found in the hair matrix (Figure 5C). The distribution pattern of proliferating cells in the skin is shown in Figure 6.

### 3.5 Monthly variation in GSDMA protein expression in skin tissue

**Figure 7:**
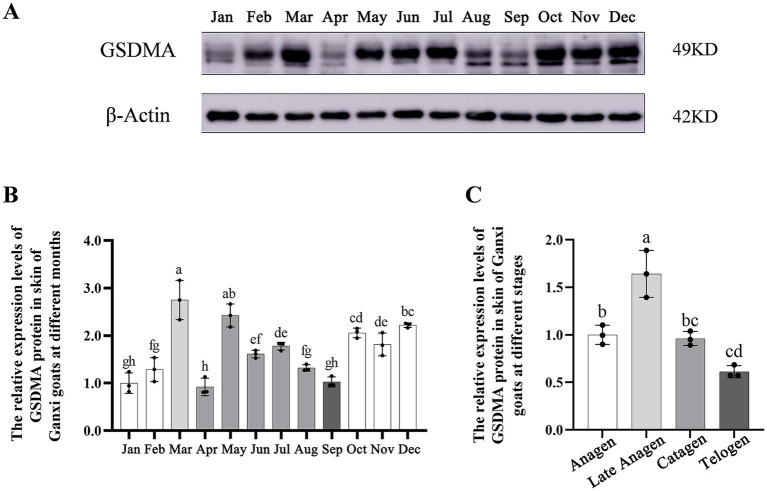
The expression changes of GSDMA protein in the skin tissue of Ganxi goats from January to December. **(A)** WB bands of GSDMA and internal reference beta-Actin in skin tissue of Ganxi goats from January to December; **(B)** The relative expression of GSDMA in skin tissue of Ganxi goats from January to December; **(C)** The relative expression of GSDMA in skin tissues of Ganxi goats at different periods. Different letters indicated a significant difference between the two groups (*p* < 0.05), and any identical letter indicated no significant difference between the two groups (*p* ≥ 0.05).

The expression level of GSDMA protein in the hair follicles of Ganxi goat skin varied across the 12 months, showing a fluctuating pattern (Figure 7A). From January to March, GSDMA expression gradually increased, followed by a decrease in April, a rise in May, and another decline in June. Between June and August, expression levels increased slowly, dropped again in September, rose in October, decreased in November, and increased once more in December. Among all months, GSDMA expression peaked in March and reached its lowest level in June. Statistical analysis showed that, except for the pairs of January–February, July–August, and October–November, GSDMA protein expression differed significantly between adjacent months (Figure 7B). In this study, the secondary hair follicle growth cycle of Ganxi goats was divided into four stages: anagen, late anagen, catagen, and telogen. GSDMA protein expression showed significant differences across these stages. From the anagen to the late anagen stage, GSDMA expression increased significantly. From late anagen through catagen to telogen, GSDMA expression showed a continuous decline. Except for the comparison between catagen and telogen, all other pairwise comparisons showed significant differences. Notably, GSDMA expression in the telogen stage was significantly lower than that in the anagen stage (Figure 7C).

### 3.6 Localization and temporal expression patterns of GSDMA protein in skin tissue

**Figure 8:**
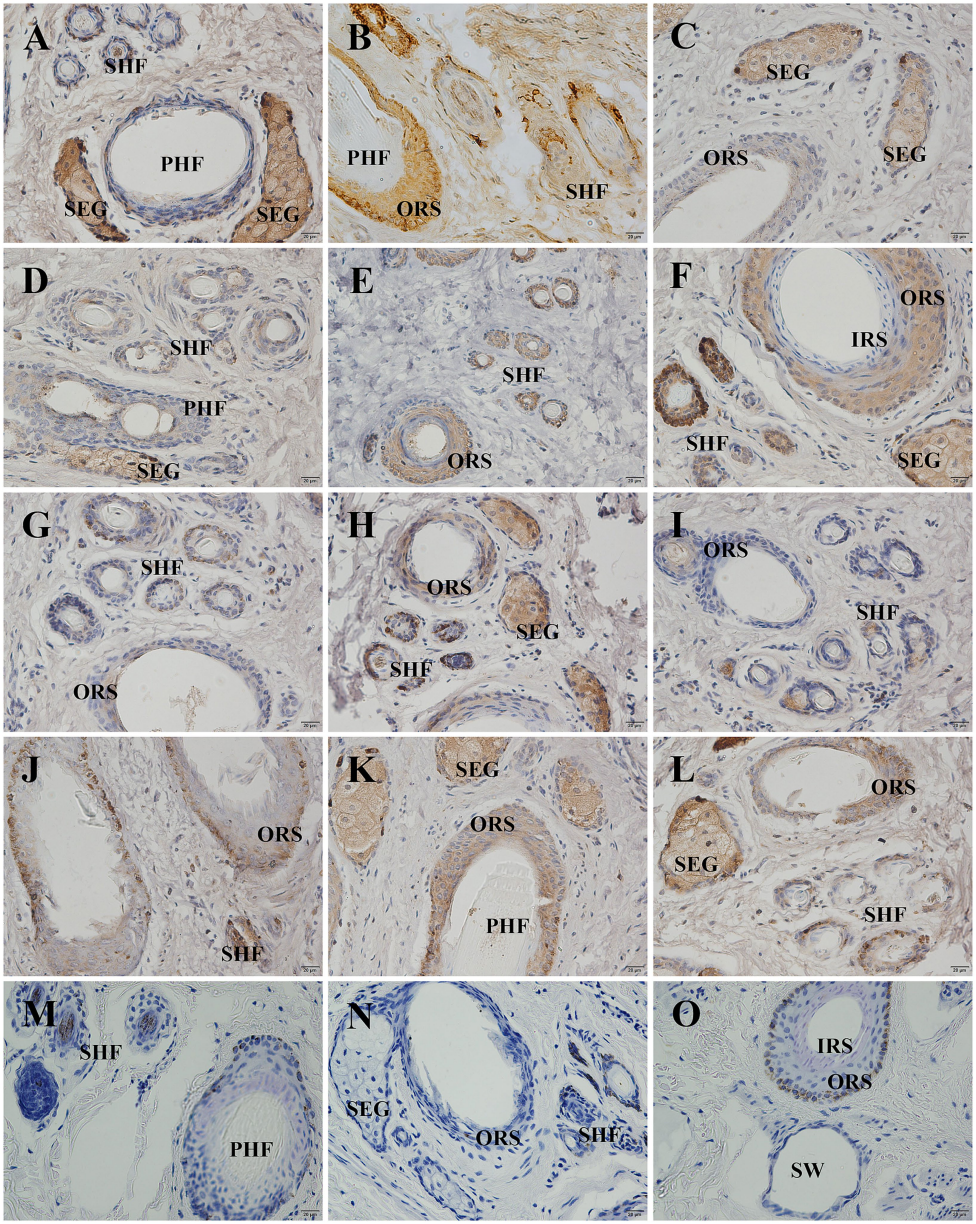
Distribution of GSDMA in individual skin tissue of Ganxi goats from January to December (cross section). **(A–L)** The skin cross section of Ganxi goat from January to December, 400×. **(M–O)** Negative control, 400×. PHF, primary hair follicle; SHF, secondary hair follicle; SEG, sebaceous glands; IRS, inner root sheath; ORS, outer root sheath; SW, sweat gland.

**Figure 9:**
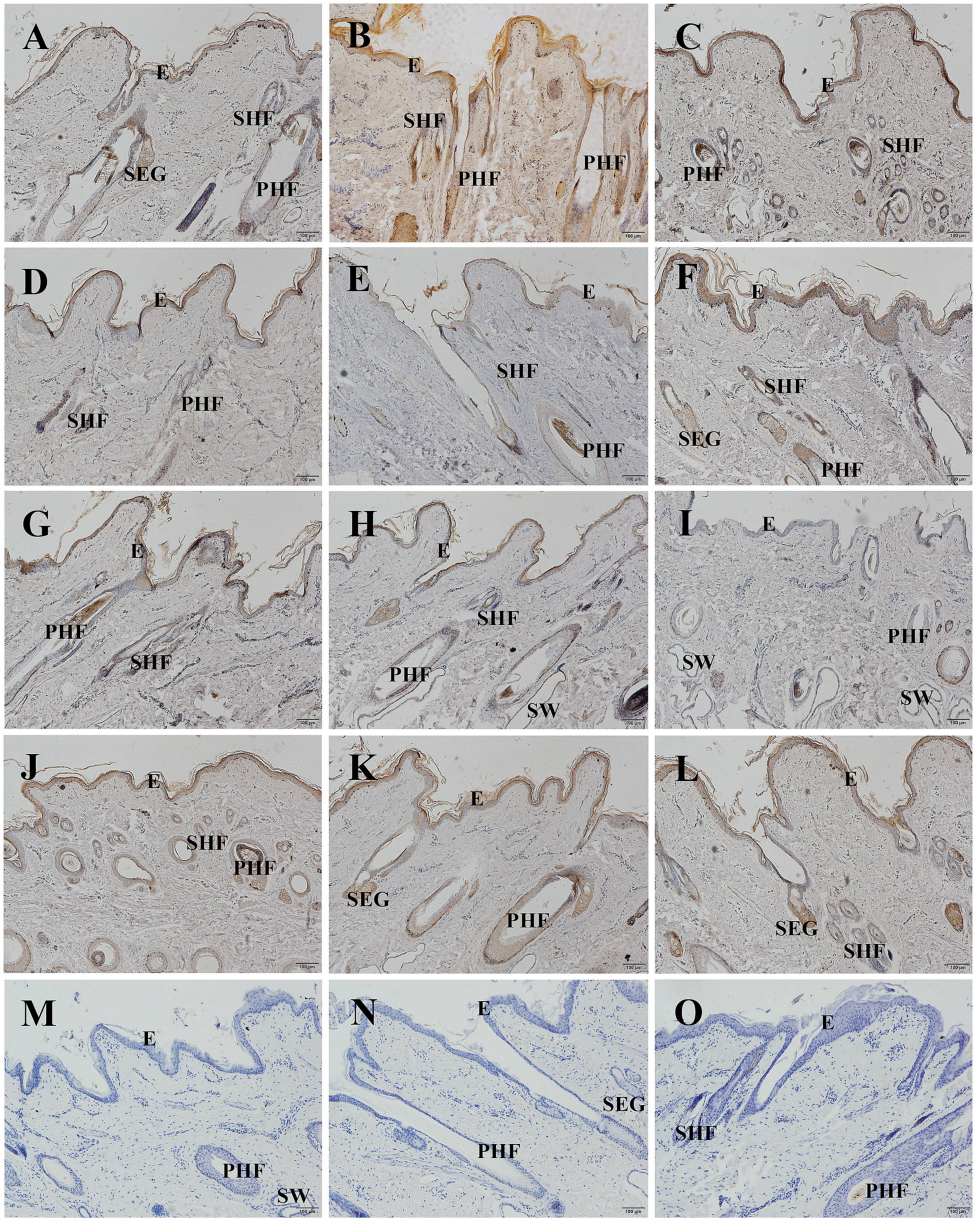
Distribution of GSDMA in individual skin tissue of Ganxi goats from January to December longitudinal (longitudinal section). **(A–L)** Ganxi goat skin longitudinal section from January to December, 100×. **(M–O)** Negative control, 100×. PHF, primary hair follicle; SHF, secondary hair follicle; SEG, sebaceous glands; SW, sweat gland.

The localization and expression patterns of GSDMA protein in skin tissue were observed under a light microscope. GSDMA-positive staining was cytoplasmic and mainly distributed in the epidermis, primary hair follicles, secondary hair follicles, and sebaceous glands. The distribution and staining intensity of GSDMA-positive cells varied across different months. In January, GSDMA was moderately expressed (yellow staining), primarily in sebaceous gland cells, inner and outer root sheaths of primary follicles, inner and outer root sheaths of secondary follicles, and the epidermis. In February, strong expression (brown-yellow staining) was observed in sebaceous gland cells, inner and outer root sheaths of primary follicles, outer root sheaths of secondary follicles, the epidermis, and the subcutaneous layer. In March, moderate expression (yellow staining) was found in sebaceous gland cells, outer root sheaths of primary and secondary follicles, the epidermis, dermis, and subcutaneous tissue. In April, moderate expression (yellow staining) was noted in sebaceous gland cells, inner and outer root sheaths of primary follicles, outer root sheaths of secondary follicles, and the epidermis, with weak expression also observed in the inner root sheath of secondary follicles. In May, weak expression (light yellow staining) was mainly found in sebaceous gland cells, outer root sheaths of primary and secondary follicles, with sparse expression in the epidermis. In June, strong expression (brown-yellow staining) appeared in sebaceous gland cells, outer root sheaths of primary and secondary follicles, and the epidermis, with limited expression in primary follicles. In July, weak expression (light yellow staining) was observed in sebaceous gland cells, outer root sheaths of primary and secondary follicles, and the epidermis. In August, moderate expression (yellow staining) was found in sebaceous gland cells, outer root sheaths of primary follicles, and the epidermis, with limited distribution in sweat glands and subcutaneous tissue. In September, weak expression (light yellow staining) was noted in sebaceous gland cells, outer root sheaths of primary and secondary follicles, and the hair cortex and medulla, with sparse expression in the epidermis. In October, moderate expression (yellow staining) was found in sebaceous gland cells, outer root sheaths of primary and secondary follicles. In November, moderate expression (yellow staining) appeared in sebaceous gland cells, inner and outer root sheaths of primary follicles, outer root sheaths of secondary follicles, and the epidermis. In December, strong expression (brown-yellow staining) was detected in sebaceous gland cells, inner and outer root sheaths of primary follicles, outer root sheaths of secondary follicles, and the epidermis, with sparse expression in the connective tissue sheath of primary follicles (Figures 8, 9).

**Figure 10:**
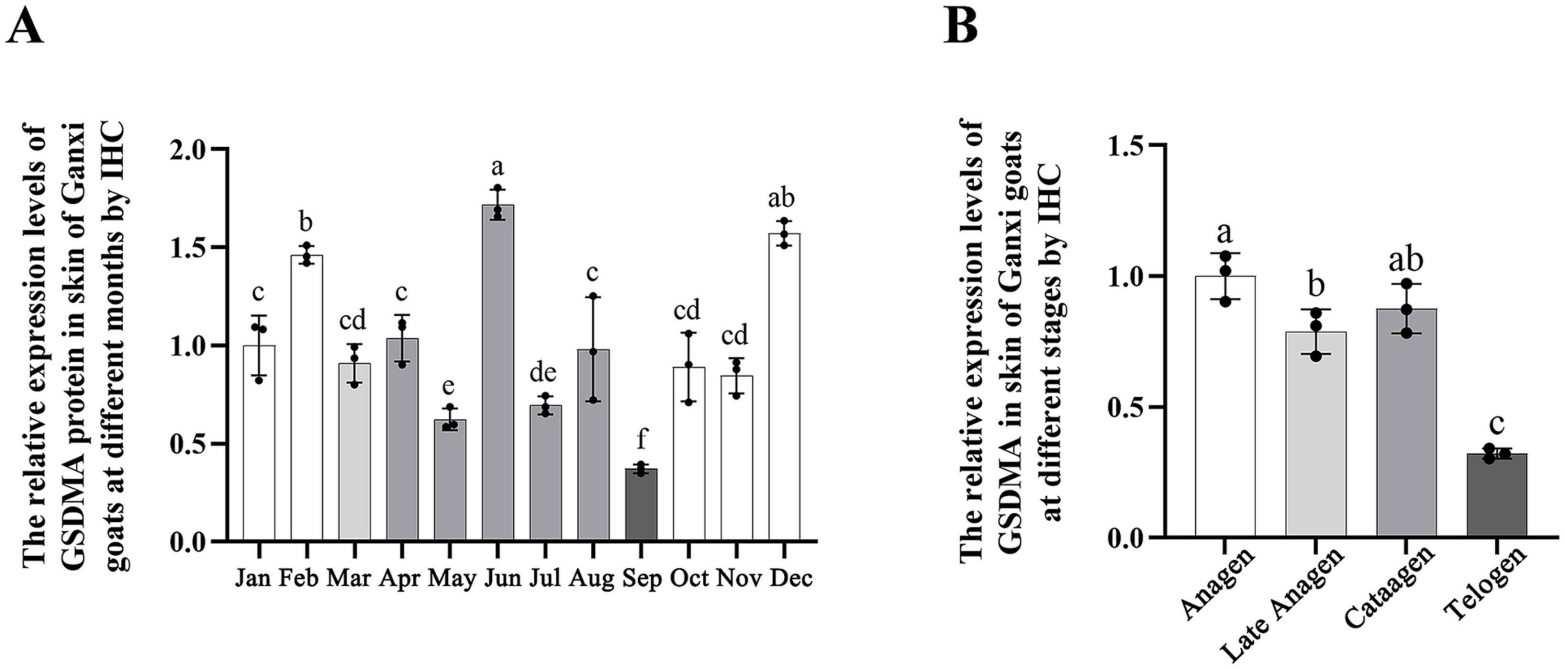
The expression changes of GSDMA in the skin tissue of Ganxi goats from January to December. **(A)** The relative expression of GSDMA in skin tissue of Ganxi goats from January to December; **(B)** The relative expression of GSDMA in skin tissues of Ganxi goats at different periods. Different letters indicated a significant difference between the two groups (*p* < 0.05), and any identical letter indicated no significant difference between the two groups (*p* ≥ 0.05).

The average optical density (AOD) of GSDMA-positive signals in skin tissue was quantified using ImageJ software, and the results are shown in Figure 10. The expression level of GSDMA protein in Ganxi goat skin varied across the months, showing a fluctuating trend. From January to February, GSDMA expression gradually increased, decreased in March, increased again in April, declined in May, and then sharply increased in June. A decrease was observed in July, followed by an increase in August, another decline in September, an increase in October, a slight decrease in November, and a final increase in December. The highest expression was recorded in June, while the lowest was observed in September. Except for the comparisons between March–April and October–November, the differences in GSDMA expression between adjacent months were statistically significant (Figure 10A). Significant differences in GSDMA protein expression were also observed among different stages of the hair follicle cycle. From the anagen to the late anagen and catagen phases, GSDMA expression first decreased and then increased. The difference between the catagen phase and both the anagen and late anagen phases was not statistically significant, while a significant difference was found between the anagen and late anagen stages. Compared with the telogen phase, both the anagen and catagen phases showed significantly higher expression levels (Figure 10B).

## 4 Discussion

As a critical organ responsible for sensing the external environment, maintaining water and heat balance, and providing barrier protection, the skin exhibits distinct structural characteristics across different species and ecological adaptation types. In this study, a systematic investigation was conducted on the skin of Ganxi goats, focusing on regional histomorphology, hair follicle structure, melanin distribution, cellular proliferation activity, hair follicle cycling, and the spatial–temporal expression of GSDMA protein. The findings aim to provide a theoretical basis for understanding the mechanisms underlying the adaptation of Ganxi goats to hot and humid climates and the regulatory processes of hair follicle cycling.

### 4.1 Relationship between skin thickness and thermoregulatory capacity in Ganxi goats

The results showed significant differences in total skin thickness, epidermal thickness, and primary hair follicle depth across different body regions of the Ganxi goat. Overall, areas such as the cranial region and dorsal neck exhibited thicker skin, while the inner forelimb and inner lower leg had relatively thinner skin. These regional differences may be related to local functional requirements and heat exchange demands (21). Thicker skin areas may enhance protection against mechanical stimuli and ultraviolet radiation, while thinner skin areas are more conducive to localized heat dissipation (22). Regarding the epidermis, the plantar and palmar regions showed greater epidermal thickness, which may be associated with their exposure to stronger friction and environmental contact (23). This increased thickness may also contribute to the regulation of local water evaporation and heat loss (24).

Differences in the depth of primary hair follicles reflect regional variations in follicular growth potential and structural stability (19). In regions such as the dorsal neck, primary hair follicles are located deeper within the dermis, which may help protect them from direct exposure to high external temperatures and support the sustained function of hair cover (10). In contrast, more superficially located follicles are likely to shed more readily during molting cycles or under heat stress, thereby increasing the exposed skin surface area and enhancing heat dissipation.

### 4.2 Hair follicle structural characteristics and skin heat dissipation regulation

The hair follicle groups of the Ganxi goat exhibit a typical “triple structure,” consisting of three primary hair follicles and 10–20 secondary follicles arranged in a relatively loose pattern. The spacing between follicle groups is wide, sweat glands are well developed, and the connective tissue sheath is underdeveloped. Compared with other breeds such as the Albas goat (25) and Inner Mongolia cashmere goat (26), the loosely arranged follicle group structure in Ganxi goats may promote better skin ventilation and thermal exchange, thereby enhancing adaptation to hot and humid environments. Regarding sebaceous gland distribution, each primary hair follicle is associated with a well-developed bilobed sebaceous gland, while secondary follicles exhibit relatively underdeveloped sebaceous glands. The secretion from sebaceous glands can form a lipid film on the hair shaft, which not only prevents water loss but also plays a role in regulating thermal conductivity to some extent (27). The degree of sebaceous gland development and its distribution pattern in Ganxi goats may reflect a dual regulatory mechanism for maintaining skin moisture and providing adequate insulation under thermal stress.

Sacpic staining further revealed the activity status of hair follicles. By staining the inner root sheath, it was possible to distinguish between the anagen, catagen, and telogen phases of the hair cycle (18). Observations showed that the primary hair follicles in Ganxi goats exhibit relatively independent cycles, with even the primary follicles within the same follicle group often existing at different stages. In some areas, dynamic replacement of old primary follicles by newly formed ones was observed. In contrast, the secondary hair follicles displayed a more synchronized cyclical turnover, although not completely synchronous. Even during the telogen phase, individual secondary follicles exhibited signs of activity. These findings suggest that Ganxi goats possess a certain regenerative capacity of hair follicles, which serves as the basis for maintaining a healthy fleece and for effective regulation of body surface temperature.

### 4.3 Melanin distribution and ultraviolet defense mechanism

The distribution of melanin on the surface of animals is not only related to coat color but also directly affects the skin’s ability to absorb and block ultraviolet (UV) radiation (28). In this study, melanin was mainly distributed in the hair bulb, outer root sheath, and hair cortex, while no obvious deposition was observed in the epidermis. This pattern contributes to enhanced photoprotection and anti-aging properties of the hair, which positively impacts fleece quality and color retention. It is consistent with the phenotypic characteristics of Ganxi goats, which exhibit white skin and black hair, and aligns with the findings of Nishimura on the mechanisms of melanocyte migration and localization (29). This distribution pattern suggests that the skin of Ganxi goats achieves UV radiation shielding primarily through increased melanin accumulation in the hair follicle system, thereby reducing the risk of DNA damage in epidermal cells and preserving the structural integrity of the skin.

### 4.4 Ki67 expression and skin renewal capacity

Ki67 is an important nuclear antigen that reflects the active state of the cell cycle, being expressed in all active phases of the cell cycle (G1, S, G2, and M) but absent in resting cells (G0), and its positive expression is commonly used to assess tissue proliferative activity (30). In the present study, Ki67 immunostaining was most prominent in the hair matrix region, consistent with the high proliferative activity of matrix keratinocytes during anagen. However, Ki67 is a general proliferation marker and does not exclusively identify matrix cells. Local sebaceous gland epithelial cells also exhibited a certain level of Ki67 positivity, indicating their proliferative capacity. Additional Ki67-positive nuclei were also detected in the basal epidermis, sebaceous glands, and outer root sheath, reflecting their normal turnover. Thus, while the observed Ki67 expression pattern supports follicular activity, it should be interpreted cautiously. Further validation with proliferation assays such as BrdU incorporation or with matrix-specific markers would be required to confirm selective activation of the follicular matrix compartment. This result aligns with the expression patterns of Ki67 and BrdU as described by Pearton et al. (31), Oshima et al. (32) and Rahmani et al. (33), reflecting active tissue renewal dynamics in different areas of goat skin. It also provides clues for further research on hair growth regulation and regeneration mechanisms. This high level of cellular turnover helps the Ganxi goat skin to rapidly repair environmental damage, maintain barrier function, and stabilize the hair follicle cycle, thus enhancing its physiological adaptability in high-temperature and high-humidity climates.

### 4.5 Characteristics of the secondary hair follicle cycle in Ganxi goats

The results of this study indicate that secondary hair follicles in the skin of Ganxi goats exhibit a distinct cyclical pattern. Based on morphological changes in follicular structures, the hair follicle cycle can be divided into four stages: anagen (October to February of the following year), late anagen (March), catagen (April to August), and telogen (September). During the anagen phase, follicle clusters appear dense, with clearly defined inner and outer root sheaths and fully developed hair shafts; secondary follicles are active and abundant. In the late anagen stage, follicular activity begins to decline, and some follicular structures become thinner. In the catagen phase, hair shafts gradually shed, follicular clusters become loose, and both the inner and outer root sheaths become thinner. By the telogen phase, most follicles are in a regressed state, with nearly absent red-stained inner root sheaths and prominent hair shaft loss, accompanied by a sharp reduction in the number of active follicles. This cyclical pattern is similar to that observed in other Chinese goat breeds, such as the Albas goat (25) and the Cashmere goat (34). However, Ganxi goats also demonstrate unique growth rhythms, such as differences in the timing of each phase, which may be closely related to breed-specific characteristics, environmental conditions, genetic background, and adaptive regulatory mechanisms (35). Recent transcriptomic studies reinforce the seasonal regulation of follicle cycling in goats. Yang et al. showed periodic gene expression changes in cashmere follicle transitions (34). Kul et al. reported seasonal expression shifts in follicle-related genes in Angora goats, directly linking gene activity to environmental conditions (36). These findings suggest that Ganxi goat follicle cycles, while observed histologically here, are likely governed by a complex interplay of genetic and epigenetic regulators responsive to environmental cues.

### 4.6 Analysis of GSDMA expression changes during different hair follicle cycle stages

GSDMA, a member of the gasdermin (GSDM) family, is widely expressed in epithelial tissues such as the skin and gastrointestinal tract (37). It plays an important role in regulating cell fate (38), maintaining epithelial integrity (13), and participating in skin-related diseases (39). In this study, the expression levels and distribution of GSDMA protein in the skin tissue of Ganxi goats were examined by Western blotting and immunohistochemistry. The results revealed distinct dynamic expression patterns of GSDMA during the hair follicle cycle, closely associated with follicular activity.

Western blot analysis showed fluctuating GSDMA protein expression across different months, with the highest levels observed in March and June. In contrast, a sharp decline was noted between June and September. From the perspective of hair follicle stages, GSDMA expression increased during the anagen phase, peaked in the late anagen stage, and gradually declined throughout the catagen and telogen phases. Statistical analysis confirmed that the majority of comparisons between phases were significantly different, suggesting that GSDMA expression is regulated by the cyclical status of the hair follicle. Although GSDMA expression generally followed the follicular cycle, with higher levels in anagen and reduced levels in catagen and telogen, monthly fluctuations were observed, including a transient peak in June during catagen. This discrepancy suggests that, beyond the follicular stage itself, other factors such as temperature, photoperiod, or endocrine status may transiently modulate GSDMA expression. Therefore, while our findings support a cycle-associated pattern, they also highlight the need for functional studies to disentangle the influence of environmental and physiological factors.

Immunohistochemical results further supported these findings. GSDMA was mainly localized in the cytoplasm and distributed throughout the epidermis, secondary and primary hair follicles, and sebaceous glands. Strong expression was particularly observed in the outer and inner root sheaths of active secondary follicles and in sebaceous gland cells. Julia et al. also demonstrated a similar tissue localization pattern of GSDMA in healthy skin using IHC techniques (15). In this study, positive GSDMA immunostaining signals were prominent during the anagen and partially in the catagen phases, but were markedly reduced or absent during the telogen phase. These results suggest that GSDMA may be involved in the maintenance of follicular activity and the regulation of hair follicle growth (40). The dynamic expression of GSDMA protein not only closely corresponds to the hair follicle cycle in Ganxi goats, but also displays distinct spatial distribution across various tissue structures, suggesting its potential involvement in multiple biological functions during follicular cycle regulation. However, its precise molecular mechanisms and direct role in environmental adaptation require further validation through additional experimental approaches.

## 5 Conclusion

Through histological observation, immunohistochemical staining, and protein expression analysis, this study revealed the morphological characteristics of hair follicle groups, the cyclic changes of secondary hair follicles, and the expression patterns of GSDMA protein at different stages of the hair follicle cycle in Ganxi goat skin. The results showed that Ganxi goats possess loosely organized follicle groups, deeply distributed hair follicles, significant regional variation in skin thickness, and a certain degree of cellular proliferative capacity. These features likely contribute to their adaptation to the hot and humid climate of western Jiangxi, providing a morphological basis for their heat tolerance and the economic value of their skin and hair. The secondary hair follicle cycle was divided into four stages: anagen (October to February), late anagen (March), catagen (April to August), and telogen (September). GSDMA protein expression was higher during anagen and late anagen, decreased during catagen, and was lowest during telogen. GSDMA was mainly localized in the cytoplasm, with strong expression in the epidermis, hair follicles, and sebaceous glands, and its expression level was closely associated with follicular activity. Beyond describing structural and molecular features, these findings provide potential markers (Ki67, GSDMA) for evaluating follicle activity and breed-specific adaptation. Future functional studies are required to clarify whether GSDMA directly contributes to thermotolerance. Such insights may ultimately inform breeding programs aimed at enhancing resilience of local goat breeds to hot and humid climates.

## Data Availability

The raw data supporting the conclusions of this article will be made available by the authors, without undue reservation.
